# Influence of Acute and Chronic Load on Perceived Wellbeing, Neuromuscular Performance, and Immune Function in Male Professional Football Players

**DOI:** 10.3390/sports13060176

**Published:** 2025-05-31

**Authors:** Alastair Harris, Tim J. Gabbett, Rachel King, Stephen P. Bird, Peter Terry

**Affiliations:** 1 AFC Bournemouth, Dorset BH7 7AF, UK; 2 School of Health and Medical Sciences, University of Southern Queensland, Ipswich 4305, Australia; stephen.bird@unisq.edu.au; 3 Gabbett Performance Solutions, Brisbane 4011, Australia; tim@gabbettperformance.com.au; 4 School of Mathematics, Physics and Computing, University of Southern Queensland, Toowoomba 4350, Australia; rachel.king@unisq.edu.au; 5 School of Psychology and Wellbeing, University of Southern Queensland, Toowoomba 4350, Australia; peter.terry@unisq.edu.au

**Keywords:** acute workload, chronic workload, load-response, adaptation

## Abstract

Objectives: The purpose of the present study was to investigate the relationship between acute and chronic loads, and the fatigue response within male elite professional football players. Design: 40-week longitudinal study across the 2021–2022 season in the English Championship. Methods: Twenty-three outfield football players had workload measured using global positioning system (Distance, High-Intensity Distance and Sprint Distance) and perceived exertion. Load-response was measured via a perceived wellbeing questionnaire, counter-movement jump (CMJ) and salivary immunoglobulin A. Results: General estimating equation models identified 18 significant interactions between workload and load-response markers. Thirteen significant interactions were found between acute and chronic workloads and CMJ variables, jump height, eccentric duration and flight contraction time. A poor CMJ was observed when acute sprint workload was >+1 standard deviation and chronic distance increased. However, when chronic perceived exertion increased, and acute sprint workload was >+1 standard deviation an advantageous response was detected on counter movement jump variables. The S-IgA response to acute and chronic workload was more variable; when chronic loads were >+1 standard deviation above mean values and acute workload increased, salivary immunoglobulin A was both suppressed and elevated depending on the interacting acute variable. Higher chronic workload was associated with better perceived wellbeing, even when acute workload was >+1 standard deviation above the mean. Conclusion: In general, low chronic workloads and acute spikes in workload were associated with poorer neuromuscular and immune function. Furthermore, CMJ performance and perceived wellbeing improved when chronic workloads were higher, despite the occurrence of acute spikes in workload.

## 1. Introduction

The demanding fixture schedule of male, English championship football, requires teams to play an average of 1.3 games per week across a 40-week season, with recovery times between games as short as two to four days [1]. Players need to tolerate such physiological and psychological stress (often referred to as ‘workload’) and be highly conditioned to physically thrive during match-play and accelerate recovery between games. The volume and variability of weekly workload across the competitive season may result in negative psycho-physiological stress responses, such as mood decrements, increased soreness, and sleep disturbances [2]. This can be influenced by fixture times, result outcomes, team selection and physical output. Sensitive monitoring strategies that encompass workload and individual load-response markers are fundamental to make well-educated decisions on player health status, preparedness, and subsequent training prescription.

The monitoring of player workloads is common practice within male professional football and is often quantified using external (i.e., the training stimulus prescribed to the athlete) and internal (i.e., measures of the athlete’s response to the exercise stressor) load [3]. Training represents a balance between overall risk (e.g., increased injury risk) and reward (e.g., improved physical qualities). This is an important consideration as training loads can be easily mismanaged and may contribute to increased fatigue and under-performance [4]. Rapid increases in workload (e.g., from week-to-week) can place acute stress on an individual, accentuating fatigue, reducing preparedness, and increasing soft tissue injury risk [4]. Previous literature within male professional football has evaluated different forms of workload analysis, including week to week variation, mesocycle analysis and differences in competitive match minutes within a week [5].

The acute-chronic workload ratio (ACWR) was introduced into the literature by Hulin, Gabbett, Blanch, Chapman, Bailey, and Orchard [6] as a modification to the Banister fitness/fatigue model, highlighting acute changes in workload in a ratio form. Chronic workloads represent fitness and acute workloads represents fatigue status. Since its introduction, a plethora of studies have used the concept to analyse changes in workload and injury association [7]. In male youth football, spikes in acute high-intensity running distance were associated with a greater relative risk of injury (RR = 2.6) when chronic workload was low compared to higher chronic loads (RR = 0.5) [8]. The positive effects of systematically progressed chronic loads are well documented, although there has been debate in the literature regarding the association between ACWR and injury risk [9]. In male professional football, high chronic workloads and well-developed physical qualities have been shown to mitigate injury risk during periods of high acute workload [10,11], and support the theoretical concept that well developed chronic loads result in a better readiness to perform and mitigate the risk of acute fatigue induced spikes in load [12].

Support staff can proactively prepare for these acute spikes in load by progressing to higher chronic workloads or by adjusting workloads when athletes experience significant reductions in wellbeing and other markers of health. There is no clear consensus on the optimal training load for performance [13,14] in professional male football, although low chronic workload and poor aerobic fitness have been shown to increase injury risk [11]. Furthermore, large variations are shown between matches for all physical metrics, with mean between-match variation in number of sprints ranging from 24–36% depending on playing position [15]. This can create unforeseen changes in weekly workload based on match selection or physical performance [16], especially if chronic workloads are lower and under preparing players for the game demands. Thus, the assessment of the physiological response to loading is vital to ensure players are well adapted and provide information on the best training prescription to optimize performance [17].

Various monitoring strategies have been used in male professional football to provide insight into the fatigue status of players and guide practitioners when making decisions regarding training prescription [2]. The mixed metabolic nature of male professional football and the high weekly muscular and metabolic demands have been shown to attenuate the neuromuscular, immunological and perceived wellbeing response after competition [18]. Practitioners require a holistic overview of the different fatigue responses, encompassing hormonal, neuromuscular and subjective measures. The Hooper index [19], salivary immunoglobulin A (s-IgA), and counter-movement jump (CMJ) analysis have all been validated as sensitive fatigue markers within male professional football [2,20]. However, no study has concurrently examined the relationships among acute and chronic workloads and perceived wellbeing, neuromuscular performance, and immune function in professional football players across a whole season.

Although several studies have investigated the relationship between acute and chronic loads and injury, few have studied the link between loading profiles and markers of fatigue. The purpose of the present study was to investigate the relationship between acute and chronic loads, and the fatigue response within male elite professional football players. It was hypothesized that low chronic loads and spikes in acute load would be associated with greater fatigue.

## 2. Materials and Methods

### 2.1. Participants

Twenty-three elite level, male, outfield football players, competing in the 2nd tier of English professional football (mean ± SD age: 24.6 ± 3.6 yr, height: 182.8 ± 7.3 cm, body mass: 78.7 ± 6.2 kg, International Society for the Advancement of Kinanthropometry 8 site skinfold sum: 42.5 ± 5.2 mm, 1600 m time trial, maximal aerobic speed: 4.9 ± 0.3) were studied across the 2021–2022 season (40 weeks). For inclusion in the study, players were required to be contracted to the Championship team’s squad. All participants received information about the study and were informed that they could withdraw at any time. After reading the participant information sheet, players provided voluntary written informed consent. The research received approval from the University of Southern Queensland ethics committee (Ethics approval #H21REA229).

### 2.2. Study Design

All data collection procedures were part of the players’ daily and weekly training practice at the club. Individual familiarization and education sessions were held with each player as part of normal club practice before any data collection. A 10-month (August 2021–May 2022) longitudinal cohort study design was used to collect s-IgA, CMJ, perceived wellbeing, and workload data. Workload data were collected from all players, whereas objective and subjective markers of fatigue were only taken for starting players of the subsequent game week during weeks when there was no midweek match. The objective and subjective markers were collected after a team day off and more than 40 h after a competitive match. Weekly training periodization was consistent throughout the season when there was seven days between matches (Game-5 Aerobic focus/Game-4 Intensive/Game-3 Extensive/Game-2 Recovery & technical/Game-1 Prep & potentiation).

### 2.3. Workload Quantification

Total distance, high-intensity distance (>19.8 km/h) and sprint distance (>25.2 km/h) were collected for all training sessions and competitive games. For all training sessions, external load variables were collected via global positioning system (GPS) technology (10 Hz, Optimeye S7, Catapult Innovations, Melbourne, Australia). GPS has been shown to provide valid and reliable measures of total, high-intensity, and sprint distances [21]. The GPS units were positioned between the players’ scapulae housed in a vest. To avoid inter-unit variation [22], each player was assigned a GPS unit at the start of the season. Across the season 16 individual player training sessions were missing due to technical reasons. In these instances, a positional average was used. Video-based time-motion analysis was used for competitive match-play (5th Generation, Tracab, Chyronhego, NY, USA). Three football clubs did not allow Tracab into their stadiums and where this occurred, average match demands from each individual were used. A global rating of perceived exertion (RPE) was collected as the internal load measurement for training and match-play [23]. It was collected individually, without words, with the player pointing to the rating on a laminated sheet of paper so other players were not influenced. This was taken 10 min after each training or match within the player dressing room. Session-RPE (s-RPE) was used to quantify internal training load, calculated by multiplying the whole training or match-play RPE using the category ratio scale (CR10-scale) [24] by the duration of the session.

### 2.4. Workload Model

Acute workload was calculated as the total accumulated load from a 7-day period (from Monday to Sunday). Chronic workload was calculated as the rolling weekly average workload from the previous four training weeks. Four external and internal load variables (total distance, high-intensity distance, sprint distance, and s-RPE) were used to assess the relationship between workload status and fatigue response. Data from the pre-season period were not included in the analysis, and load-response markers were only taken from players who were participating in full team training during the previous week.

### 2.5. Saliva Testing Protocol

A total of 408 saliva samples were collected at the same time of day each week (between 09:00–09:30 a.m.) and analyzed for s-IgA. The collection and analysis of the saliva samples occurred as described and validated previously within professional football [25], and validated against enzyme-linked immunosorbent assay (ELISA) (R^2^ = 0.78) [26]. Volume and intensity of exercise have been shown to either suppress or elevate the immune response [20,27], providing justification of a sensitive fatigue response marker within professional football. Four baseline measures were taken for each player, at least 60 h after their last exercise exposure, whereby an individual’s mean s-IgA was determined by averaging all data points.

### 2.6. Countermovement Jump Testing Protocol

Counter-movement jump (CMJ) testing was performed at the same time of day (between 10:00–10:30 a.m.) at least 60 h after the last exercise exposure. All participants completed a standardized 20-min warm up protocol before testing, as part of their daily movement preparation for training. Before each jump, participants were instructed to stand still and have their body mass taken, with each foot positioned on a portable vertical axis force plate (35 × 70 cm, FD4000, ForceDecks, VALD Performance Pty Ltd., Brisbane, Australia) that simultaneously sampled at 1000 Hz. The portable force plate has previously been validated against laboratory ground-based force platform systems [28]. The participants performed three maximal jumps interspersed by 20 s of recovery between each effort, aiming to jump as high as they could with a fast counter movement phase from a self-selected depth. Jump height (cm) (coefficient of variation (CV) = 5.4%), eccentric duration (ms) (CV = 11.7%), eccentric mean power (W/Kg) (CV = 17.9%), and flight:contraction time (FT:CT) (CV = 9.3%) [29] were measured and the mean value was taken for each variable. Variables were selected to give analysis across the force velocity curve, encompassing the eccentric and concentric demands of the CMJ as per Harper, Cohen, Carling, and Kiely [30] Three reference range measures were taken for each player across the season at set time points (Week 1, Week 23, Week 43). The mean of the three best jumps was calculated and used as the individuals reference range. This was performed at least 60 h after any physical activity.

### 2.7. Perceived Wellbeing

Perceived wellbeing was collected daily, upon arrival to the team dressing room at the training ground, via a psychometric questionnaire [19]. The data were collected individually, with a staff member present and data was entered into an iPad. The questionnaire assessed players’ sleep quality, fatigue, muscle soreness, mood, and stress, on a seven-point Likert scale (1–7) [19] For subjective load-response assessment of the previous week’s load, the sum of daily wellbeing scores for each individual was taken on the first training day of the following week, after a team day off, and more than 40 h post competitive match.

### 2.8. Data Analysis

Descriptive statistics including mean, standard deviations, CV, and interquartile ranges were generated for all load variables (Table 1). General estimating equations (GEE) were used to model the association between workload (4 acute and 4 chronic variables) and each of the six load-response variables in the subsequent week. The GEE method was used because it allows for the correlated and repeated nature of the data within players and for the inclusion of multiple covariates. GEEs are a nonparametric method providing population averaged estimates based on a working correlation matrix. All statistical analysis was performed using the R statistical software (R core Team, 2023, RStudio (RStudio Team, 2020, Boston, MA, USA) and the “geepack” package [31].

All eight load variables were standardized by player (value-mean of player/standard deviation of player) to adjust for the individual nature of the weekly fluctuations of workload. All load-response variables were continuous measures and therefore, a Gaussian link function was used in the GEE models. Interaction effects between continuous predictors can be difficult to interpret, subsequently we focused on linear analysis, consistent with previous research [32,33].

All main effects and 2-way interactions between acute and chronic load variables were analyzed for their relationships with each load-response variable. For any significant effects (*p* < 0.01) within the GEE models, plots were produced to show the change in the load-response variable (y-axis) associated with change in the load variable (x-axis). For significant 2-way interactions, one load variable was represented as a continuous measure on the x-axis, while the other was categorized to show the change in the interaction effect when that load variable was, (a) below −1 SD; (b) between −1 SD and +1 SD; (c) above +1 SD. The exchangeable correlation structure was determined to be the best fit for each model: For each load-response model the correlation structure was chosen by comparing models with independence, exchangeable and ar1 correlation structures. The lowest correlation information criterion (CIC) value was used to determine the best fit. CIC has been suggested as a more robust alternative to QIC which is a generalisation of Akaike’s Information Criterion (AIC) for GEE models.

## 3. Results

Descriptive statistics for each of the eight workload variables (Table 1) show that for each of the four workload types (total distance, high-intensity distance, sprint distance and RPE), the mean acute and chronic values were similar. Acute variables had larger standard deviations (SD), inter-quartile ranges (IQR) and ranges (Min-Max) than their associated chronic variables.

Fluctuations throughout the season are represented on all workload variables. The min is much lower than Q1 and the max much higher than Q3. These differences are larger than the difference between Q1 and Q3. This indicates that the middle 50% of data represents weeks with moderate variability in the workload measures, with some weeks having more extreme low or high measures. The mean and standard deviation are within the IQR and also indicates a moderate variability around the mean.

Descriptive statistics for the six load-response variables are shown in Table 2. The load-response variables were within normative ranges of CVs as reported in previous studies [25,34].

The GEE models identified 18 significant workload effects related to the load-response markers. Thirteen significant interactions were found between acute and chronic workloads and CMJ variables. s-IgA was significantly influenced by the interaction of acute and chronic workload variables and there was also an association between perceived wellbeing and acute and chronic workloads. The interactions between chronic distance and acute sprint workload and chronic RPE and acute sprint workload, influenced the highest number of load-response markers (four out of the six) and in total 10 different workload interactions influenced the load-response markers.

**Figure 1:**
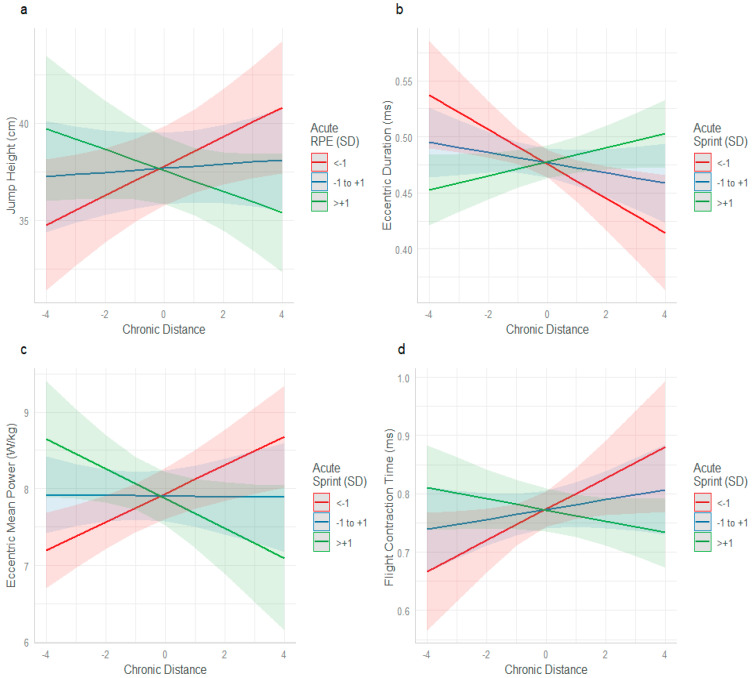
Acute and chronic training load interactions and their effect on jump height, eccentric duration, eccentric mean power and flight:contraction time performance. Interaction between acute RPE and chronic distance for: (**a**) jump height. (**b**) Interaction between chronic distance and acute sprint for: eccentric duration, (**c**) eccentric mean power and (**d**) flight contraction time.

When analysing the CMJ results, only one workload interaction, between chronic distance and acute RPE, was found to significantly affect jump height (β = −0.64857, SE = 0.25093, *p* = 0.009). When acute RPE was <−1 SD below the mean and chronic distance increased, jump height improved (red line, Figure 1a). However, when acute RPE was >+1 SD above the mean and chronic distance increased, jump height tended to decrease (green line).

The interaction between chronic distance and acute sprint was significant for the other three CMJ variables (eccentric duration, β = 0.010847, SE = 0.003074, *p* < 0.001; eccentric mean power, β = −0.19010, SE = 0.05015, *p* < 0.001 FT:CT, β = −0.018239, SE = 0.005990, *p* = 0.002). When acute sprint distance was <−1 SD and chronic distance increased, eccentric duration improved (red line, Figure 1b), whereas it was poorer when acute sprint was >+1 SD (green line). Eccentric mean power (Figure 1c) and FT:CT (Figure 1d) both improved when chronic distance increased, and acute sprint distance was <−1 SD (red line) and reduced when acute sprint was >+1 SD (green line).

**Figure 2:**
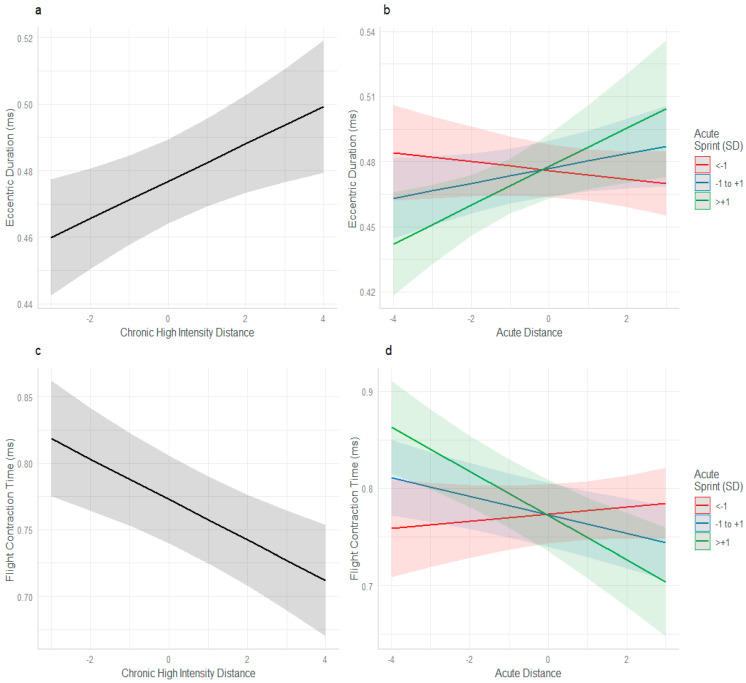
Acute and chronic training load interactions and their effect on eccentric duration and flight contraction time performance. (**a**) Interaction between chronic high-intensity distance and eccentric duration, (**b**) Interaction between acute distance and acute sprint and eccentric duration, (**c**) Interaction between chronic high-intensity distance and flight contraction time, and (**d**) interaction between acute distance and acute sprint and flight contraction time.

Chronic high-intensity distance (β = 0.005620, SE = 0.001990, *p* = 0.004), and the interaction between acute distance and acute sprint distance (β = 0.005467, SE = 0.002089, *p* = 0.008) influenced eccentric duration (Figure 2a,b). Chronic high-intensity distance (β = −0.015233, SE = 0.004052, *p* < 0.001) and the interaction between acute distance and acute sprint distance (β = −0.013180, SE = 0.003973, *p* < 0.001) influenced FT:CT.

As chronic high-intensity distance increased in isolation a significant effect was detected for both eccentric duration (slower) and FT:CT (slower). Similarly, the workload interaction of acute sprint and acute distance influenced both eccentric duration and FT:CT in the same way. When acute sprint was <−1 SD below the mean and acute distance increased, eccentric duration and FT:CT improved (red line, Figure 2b,d). However, when acute sprint was >+1 SD above the mean and acute distance increased, eccentric duration and FT:CT tended to slow (green line).

For s-IgA, interactions between acute sprint and chronic RPE (β = −0.15090, SE = 0.05450, *p* = 0.005), acute high-intensity distance and chronic distance (β = −0.17379, SE = 0.06266, *p* = 0.005), acute sprint and chronic distance (β = 0.17797, SE = 0.05559, *p* = 0.001) and acute high-intensity distance and chronic RPE (β = 0.17990, SE = 0.05133, *p* < 0.001) were found.

**Figure 3:**
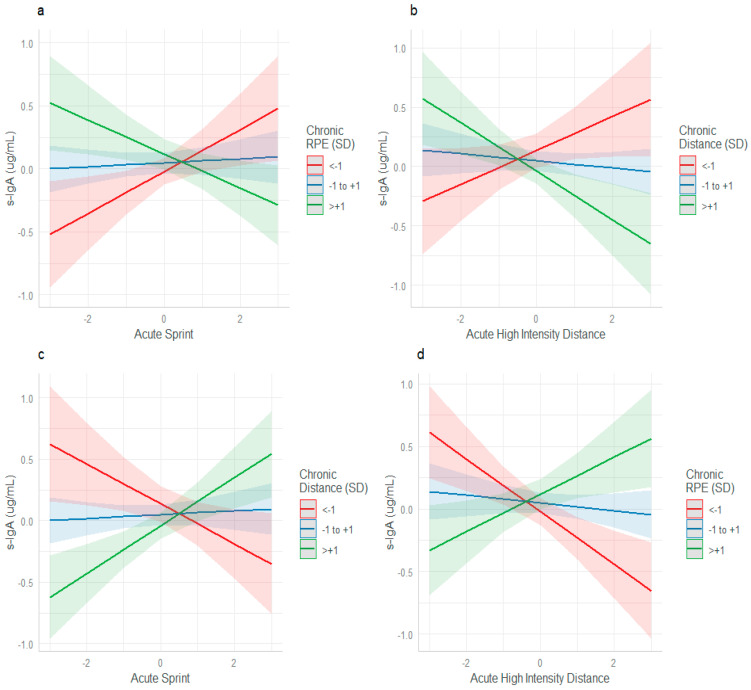
Acute and chronic training load interactions and their effect on s-IgA. (**a**) Interaction between acute sprint and chronic RPE (**b**), acute high-intensity distance and chronic distance (**c**), acute sprint with chronic distance, and (**d**), acute high-intensity distance and chronic RPE.

When chronic RPE and chronic distance were <−1 SD below the mean and acute sprint and acute high-intensity distance increased, s-IgA was elevated (red line, Figure 3a,b). However, when chronic RPE and chronic distance were >+1 SD above the mean and acute sprint and acute high-intensity distance increased, s-IgA suppression occurred (green line). When analyzing the workload interactions between chronic RPE and acute high-intensity distance and chronic distance and acute sprint the reverse effects were seen on s-IgA (Figure 3c,d). When chronic distance and chronic RPE were <−1 SD below the mean and acute sprint and acute high-intensity distance increased, s-IgA suppression was seen (red line, Figure 3c,d). However, when chronic distance and chronic RPE were >+1 SD above the mean and acute sprint and acute high-intensity distance increased, s-IgA elevation was observed (green line).

**Figure 4:**
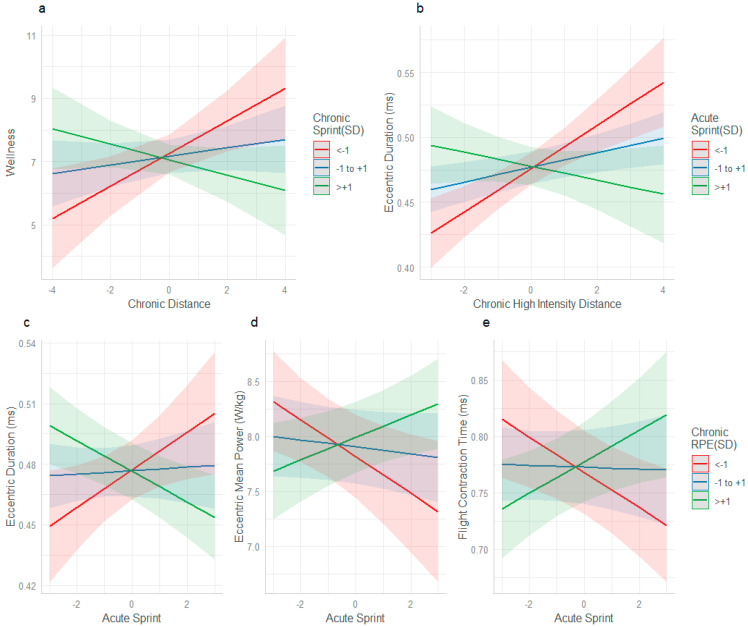
The interaction effects of chronic distance and chronic sprint on load-response markers. (**a**) Interaction between chronic distance and chronic sprint distance on wellbeing, (**b**) chronic high-intensity distance and acute sprint on eccentric duration, (**c**) acute sprint distance and chronic RPE on eccentric duration, (**d**) eccentric mean power, and (**e**) flight contraction time.

For perceived wellbeing, the interaction between chronic distance and chronic sprint distance (β = −0.0379, SE = 0.01304, *p* = 0.003) was significant, whereby, when chronic sprint was <1 SD (red line) or within mean parameters (blue line) and chronic distance increased, perceived wellbeing decreased (Figure 4a). When chronic sprint was >+1 SD (green line) and chronic distance increased, perceived wellbeing improved. This same interaction was observed between acute sprint and chronic high-intensity distance and eccentric duration (β = −0.011011, SE = 0.003754, *p* = 0.003), between acute sprint distance and chronic RPE and eccentric duration (β = −0.008477, SE = 0.002513, *p* < 0.001), eccentric mean power (β = 0.13399, SE = 0.04937, *p* = 0.006), and FT:CT (β = 0.014775, SE = 0.004250, *p* < 0.001). (Figure 4). The workload interactions between chronic RPE and acute sprint and their effect on eccentric duration, eccentric mean power and FT:CT all followed the same pattern. When chronic RPE was either <−1 SD (red line) or within mean parameters (blue line), and acute sprint increased, the effect on the load-response marker was deleterious. When chronic RPE was >+1 SD (green line) and acute sprint increased, the effect on the load-response markers was advantageous (Figure 4c–e)

## 4. Discussion

The present study investigated the influence of acute and chronic workload on perceived wellbeing, neuromuscular performance, and immune function in male professional football players over the course of a competitive season. The new findings of this study include: (1) neuromuscular and immune function were poorer when (collectively), acute loads were high and chronic loads were low, (2) neuromuscular function improved when both acute and chronic loads were high, and (3) perceived wellbeing improved when both acute and chronic loads were higher. From a practical perspective, these findings suggest a protective effect of higher chronic workloads when players are exposed to larger acute workloads.

To the best of the authors’ knowledge, this is the first study to concurrently investigate the relationships among workload, perceived wellbeing, neuromuscular performance, and immune function in male professional football players. The major finding highlights the potential protective effect of higher chronic loads when male players are exposed to acute spikes in workload. Players with higher chronic internal load (s-RPE), who were concurrently exposed to high acute sprint distances, experienced increases in eccentric mean power and improved FT:CT and eccentric durations during CMJ testing (Figure 4c–e). Consistent with our finding of the potential protective effect of higher chronic loads, male players with lower chronic loads, experienced reduced neuromuscular performance (in the form of longer eccentric duration time (Figure 1b and Figure 4b,c) lower FT:CT time (Figure 1d and Figure 4e), and lower eccentric mean power (Figure 4d) when exposed to acute spikes in both high-speed running and sprinting. These findings demonstrate that in comparison to lower chronic loads, high chronic loads may better prepare male professional football players to tolerate acute spikes in workload, while also enhancing neuromuscular function. A rationale for this was previously reported by Morgans et al. [16]. They demonstrated that higher match minutes across a season, increased weekly workloads and provided an advantageous neuromuscular stimulus in comparison with players with reduced workloads. The present study demonstrates that regular exposure to workloads that provide high neuromuscular demand, such as sprinting, provide adaptative stimulus, to enhance, and preserve these qualities during higher weekly workloads in comparison to lower chronic exposure. With lower chronic workloads, the frequency, volume, and dose, of neuromuscular training, may not be high enough to moderate the fatigue response of a spike in workload.

Our finding of suppressed s-IgA with higher acute and chronic loads is consistent with the results of other male professional football studies [25,35]. Sustained, high volume training (>+1 SD, chronic distance) suppressed s-IgA when coupled with spikes in acute high-intensity distance (Figure 3b). This was also evident when chronic RPE and acute sprint distance were high (Figure 3c). Gleeson et al. [36] reported that a suppression of s-IgA occurs during periods of high volume or high-intensity workload across a 7-month study in elite swimmers. Our study also showed that periods of high chronic distance and chronic RPE were associated with elevated s-IgA when acute spikes in sprint and high-intensity distance occurred (Figure 3b,d). These findings are consistent with the literature, validating s-IgA as a biological stress marker and a useful tool to infer immune system function [20]. There is more variance in weekly workload and training stimulus across the energy systems in male professional football when compared to elite endurance swimmers [36], who saw a transient reduction in s-IgA over a longitudinal period with high volume endurance training. Nevertheless, the findings of the present study, indicate that when workload deviates from homeostasis, either acutely or chronically, s-IgA is a sensitive tool to detect suppression of immune function.

A disruption (either elevation or suppression) in immune homeostasis can result in reduced exercise performance and diminished ability to sustain heavy training [20]. This can then compromise an athlete’s ability to prepare for their next competitive encounter, and reduce their adaptative capability from a training or competition exercise stimulus [37]. Therefore, we hypothesize that the ability to proactively plan a players’ training week based on s-IgA information can help practitioners reduce the risk of underperformance by manipulating the volume and intensity of training for the subsequent week.

The finding of an improved state of wellbeing when chronic distance and sprint workloads were high (Figure 4a) is consistent with other studies that have demonstrated that acute and chronic loads with less variation, resulted in better perceived wellbeing in male professional footballers [38]. This finding shows the value of consistent, appropriate, and well prescribed chronic workloads in improving the perceived fatigue response. Contrary to previous literature in male football [39], no significant interactions were found between acute workload spikes and increased subjective fatigue. Thorpe et al. [18] highlighted an elevated perceived fatigue response following acute spikes in high-intensity running and Moalla et al. [38] described disruptions to perceived sleep quality, when acute workloads were high. A possible explanation for the differing results between the present and previous studies is the methodology used. Both Thorpe et al. [18] and Moalla et al. [38] analyzed the individual constructs (i.e., sleep, fatigue, stress etc.) daily. Conversely, the present study measured global wellbeing on the first day of the week, after a day off. Nonetheless, the findings of Thorpe et al. [18] and Moalla et al. [38] support the main findings of the present study, describing a deviation in the fatigue response when an individuals’ workload fluctuates outside of a normal range.

The acute and chronic workloads performed by the professional football players in our study were higher than previously reported in male Premier League footballers [10]. This demonstrates that the participants in our study were exposed to a physically demanding training and match model across the season, encompassing consistently high chronic workloads. Well-developed chronic workloads represent a higher state of fitness and provide athletes with a better ability to tolerate acute spikes in workload [4], observed previously within male professional and youth football [10,11]. However, unforeseen spikes in acute workload can occur due to the variation in match-to-match physical demands or following prolonged exposure to limited playing time. In English male, professional football, the weekly match stimulus results in higher workloads for starters than substitutes [16]. Moreover, natural variation occurs in match-to-match physical load due to the differing tactical constraints, possession time, score line, weather and playing position [15], exposing the players to differing levels of physiological strain from match-to-match. There is a lack of consensus within the literature regarding an optimal acute and chronic load to adequately prepare male players for the demands of match-play [13,14]. The present study provides evidence to support the notion that having a chronic workload that enables a player to absorb the acute demands of match-play, whilst also allowing them to prepare for the most demanding passages of play, should support optimal performance and mitigate the fatigue response.

## 5. Conclusions

Eighteen significant interactions were found between workload and load-response markers. In general, when chronic workloads were low and spikes in acute workload occurred, significantly poorer neuromuscular and immune function occurred. Furthermore, CMJ performance and perceived wellbeing improved when chronic workloads were higher and acute spikes in workload occurred.

## 6. Limitations

This study has some limitations that should be recognized. Firstly, the study did not differentiate between starters and substitutes for acute and chronic workloads and their subsequent effect on the load-response markers. Therefore, the significant interactions observed between workload variables and the load-response markers do not account for how those workloads were accumulated. Secondly, the external workload was assessed using traditional speed band classification variables. Therefore, the mechanical load of acceleration, deceleration, and changes of direction, along with the metabolic stress of locomotion covered above an individual’s maximal aerobic speed or the higher ranges of their anaerobic speed reserve, were not accounted for in the workload variables. As technology advances and the ability to reliably assess these metrics in both training and match-play improves, this should be an area of focus for future research. Thirdly, there are numerous methods to assess wellbeing, neuromuscular function and immunological function. This study focused on three valid methods for assessing these parameters. Lastly, it is important to note that this study was only carried out on male professional footballers. Although inferences can be made due to the similar energetic and metabolic demands involved in male and female football, future research including both genders is warranted.

## Figures and Tables

**Table 1:** Descriptive statistics for workload variables (*N* = 696).

Variable	Mean	SD	Min	Max	IQR	Q1	Q3
Acute Distance (m)	27,027	7165	3643	48,907	9289	22,352	31,641
Chronic Distance (m)	27,015	5130	10,743	45,237	6278	24,194	30,472
Acute HI (m)	1674	682	11	3666	962	1178	2140
Chronic HI (m)	1676	539	500	3230	800	1256	2056
Acute Sprint (m)	394	218	0	1051	308	228	535
Chronic Sprint (m)	387	158	63	800	242	269	511
Acute RPE (AU)	1961	623	260	3887	764	1578	2342
Chronic RPE (AU)	1954	477	283	3549	610	1664	2274

Abbreviation: HI = High-intensity distance; RPE = Rate of perceived exertion.

**Table 2:** Descriptive statistics for load-response variables.

	Sample Size (n)	Weeks (Min, Max)	Mean ± SD	%CV (LCI, UCI)	ICC (LCI), UCI)
Wellbeing	637	(11, 39)	7.16 ± 1.91	21.2 (18.4, 23.9)	0.41 (0.25, 0.53)
s-IgA (ug/mL)	406	(7, 25)	216.38 ± 173.30	50.8 (46.2, 55.5)	0.47 (0.28, 0.59)
Ecc Duration (ms)	402	(3, 30)	0.47 ± 0.04	5.1 (4.2, 6.0)	0.55 (0.34, 0.67)
Ecc Mean Power (W/Kg)	402	(3, 30)	7.90 ± 0.88	5.7 (4.8, 6.6)	0.70 (0.52, 0.79)
FT:CT	402	(3, 30)	0.78 ± 0.09	6.1 (5.2, 7.1)	0.67 (0.48, 0.78)
Jump Height (Imp-mom, cm)	402	(3, 30)	37.96 ± 5.19	5.8 (5.0, 6.6)	0.79 (0.63, 0.86)

Abbreviations: s-IgA = Salivary Immunoglobulin A; Ecc = Eccentric; FT:CT = Flight Contraction Time.

## Data Availability

Restrictions apply to the availability of these data. Data were ob-tained from AFC Bournemouth, Bournemouth, England and are available from the corresponding author with the permission of AFC Bournemouth. The de-identification of data is not possible as it will be easy to decipher the identity of the players based around upon their position and match and training statistics from that season. This could have asset value implications that could affect the football club and the individual player. The de-identification of the players has not been authorised by AFC Bournemouth.

## References

[1] Springham M., Williams S., Waldron M., Burgess D., Newton R.U. (2020). Large reductions in match play physical performance variables across a professional football season with control for situational and contextual variables. Front. Sports Act. Living.

[2] Thorpe R.T., Strudwick A.J., Buchheit M., Atkinson G., Drust B., Gregson W. (2015). Monitoring fatigue during the in-season competitive phase in elite soccer players. Int. J. Sports Physiol. Perform..

[3] Impellizzeri F., Rampinini E., Coutts A., Sassi A., Marcora S. (2004). Use of RPE-based training load in soccer. Med. Sci. Sports Exerc..

[4] Gabbett T.J. (2016). The training—injury prevention paradox: Should athletes be training smarter and harder?. Br. J. Sports Med..

[5] Teixeira J.E., Forte P., Ferraz R., Leal M., Ribeiro J., Silva A.J., Barbosa T.M., Monteiro A.M. (2021). Monitoring accumulated training and match load in football: A systematic review. Int. J. Environ. Res. Public Health.

[6] Hulin B.T., Gabbett T.J., Blanch P., Chapman P., Bailey D., Orchard J.W. (2014). Spikes in acute workload are associated with increased injury risk in elite cricket fast bowlers. Br. J. Sports Med..

[7] Griffin A., Kenny I.C., Comyns T.M., Lyons M. (2020). The association between the acute:chronic workload ratio and injury and its application in team sports: A systematic review. Sports Med..

[8] Bowen L., Gross A.S., Gimpel M., Li F.-X. (2017). Accumulated workloads and the acute:chronic workload ratio relate to injury risk in elite youth football players. Br. J. Sports Med..

[9] Impellizzeri F., Woodcock S., Coutts A.J., Fanchini M., McCall A., Vigotsky A. (2020). Acute to random workload ratio is ‘as’ associated with injury as acute to actual chronic workload ratio: Time to dismiss ACWR and its components. SportRxiv Preprints.

[10] Bowen L., Gross A.S., Gimpel M., Bruce-Low S., Li F.-X. (2020). Spikes in acute:chronic workload ratio (acwr) associated with a 5–7 times greater injury rate in English premier league football players: A comprehensive 3-year study. Br. J. Sports Med..

[11] Malone S., Owen A., Newton M., Mendes B., Collins K.D., Gabbett T.J. (2017). The acute:chonic workload ratio in relation to injury risk in professional soccer. J. Sci. Med. Sport.

[12] Hulin B., Gabbett T., Pickworth N., Johnston R., Jenkins D. (2019). Relationships among playerload, high-intensity intermittent running ability, and injury risk in professional rugby league players. Int. J. Sports Physiol. Perform..

[13] Anderson L., Orme P., Di Michele R., Close G.L., Morgans R., Drust B., Morton J.P. (2016). Quantification of training load during one, two and three game week schedules in professional soccer players from the English Premier League: Implications for carbohydrate periodisation. J. Sports Sci..

[14] Stevens T.G.A., de Ruiter C.J., Twisk J.W.R., Savelsbergh G.J.P., Beek P.J. (2017). Quantification of in-season training load relative to match load in professional Dutch Eredivisie football players. Sci. Med. Footb..

[15] Gregson W., Drust B., Atkinson G., Di Salvo V. (2010). Match-to-match variability of high-speed activities in premier league soccer. Int. J. Sports Med..

[16] Morgans R., Di Michele R., Drust B. (2018). Soccer match play as an important component of the power-training stimulus in premier league players. Int. J. Sports Physiol. Perform..

[17] Gabbett T.J., Nassis G.P., Oetter E., Pretorius J., Johnston N., Medina D., Rodas G., Myslinski T., Howells D., Beard A. (2017). The athlete monitoring cycle: A practical guide to interpreting and applying training monitoring data. Br. J. Sports Med..

[18] Thorpe R.T., Atkinson G., Drust B., Gregson W. (2017). Monitoring fatigue status in elite team-sport athletes: Implications for practice. Int. J. Sports Physiol. Perform..

[19] Hooper S.L., Mackinnon L.T., Howard A., Gordon R.D., Bachmann A.W. (1995). Markers for monitoring overtraining and recovery. Med. Sci. Sports Exerc..

[20] Springham M., Newton R.U., Strudwick A.J., Waldron M. (2022). Selected immunoendocrine measures for monitoring responses to training and match load in professional association football: A review of the evidence. Int. J. Sports Physiol. Perform..

[21] Makar P., Silva A.F., Oliveira R., Janusiak M., Parus P., Smoter M., Clemente F.M. (2023). Assessing the agreement between a global navigation satellite system and an optical-tracking system for measuring total, high-speed running, and sprint distances in official soccer matches. Sci. Prog..

[22] Varley M.C., Jaspers A., Helsen W.F., Malone J.J. (2017). Methodological considerations when quantifying high-intensity efforts in team sport using global positioning system technology. Int. J. Sports Physiol. Perform..

[23] Foster C.C., Florhaug J.A., Franklin J., Gottschall L., Hrovatin L.A., Parker S., Doleshal P., Dodge C. (2001). A new approach to monitoring exercise training. J. Strength Cond. Res..

[24] Borg G., Hassmén P., Lagerström M. (1987). Perceived exertion related to heart rate and blood lactate during arm and leg exercise. Eur. J. Appl. Physiol. Occup. Physiol..

[25] Morgans R., Orme P., Anderson L., Drust B., Morton J. (2014). An intensive winter fixture schedule induces a transient fall in salivary IgA in English premier league soccer players. Res. Sports Med..

[26] Kremer D., Metzger S., Kolb-Bachofen V., Kremer D. (2012). Quantitative measurement of genome-wide DNA methylation by a reliable and cost-efficient enzyme-linked immunosorbent assay technique. Anal. Biochem..

[27] Neville V., Gleeson M., Folland J. (2008). Salivary IgA as a risk factor for upper respiratory infections in elite professional athletes. Med. Sci. Sports Exerc..

[28] Lake J., Mundy P., Comfort P., McMahon J., Suchomel T., Carden P. (2018). Concurrent validity of a portable force plate using vertical jump force-time characteristics. J. Appl. Biomech..

[29] Heishman A.D., Daub B.D., Miller R.M., Freitas E.D.S., Frantz B.A., Bemben M.G. (2020). Countermovement jump reliability performed with and without an arm swing in NCAA division 1 intercollegiate basketball players. J. Strength Cond. Res..

[30] Harper D.J., Cohen D.D., Carling C., Kiely J. (2020). Can countermovement jump neuromuscular performance qualities differentiate maximal horizontal deceleration ability in team sport athletes?. Sports.

[31] Højsgaard S., Halekoh U., Yan J. (2005). The R package geepack for generalized estimating equations. J. Stat. Softw..

[32] Ryan S., Crowcroft S., Kempton T., Coutts A.J. (2021). Associations between refined athlete monitoring measures and individual match performance in professional Australian football. Sci. Med. Footb..

[33] Jaspers A., Kuyvenhoven J., Staes F., Frencken W., Helsen W., Brink M. (2018). Examination of the external and internal load indicators’ association with overuse injuries in professional soccer players. J. Sci. Med. Sport.

[34] McLean B.D., Coutts A.J., Kelly V., McGuigan M.R., Cormack S.J. (2010). Neuromuscular, endocrine, and perceptual fatigue responses during different length between-match microcycles in professional rugby league players. Int. J. Sports Physiol. Perform..

[35] Owen A.L., Wong D.P., Dunlop G., Groussard C., Kebsi W., Dellal A., Morgans R., Zouhal H. (2016). High-intensity training and salivary immunoglobulin a responses in professional top-level soccer players: Effect of training intensity. J. Strength Cond. Res..

[36] Gleeson M., Mcdonald W.A., Cripps A.W., Pyne D.B., Clancy R.L., Fricker P.A. (2008). The effect on immunity of long-term intensive training in elite swimmers. Clin. Exp. Immunol..

[37] Malm C., Ekblom O., Ekblom B. (2004). Immune system alteration in response to two consecutive soccer games. Acta Physiol. Scand..

[38] Moalla W., Fessi M.S., Farhat F., Nouira S., Wong D.P., Dupont G. (2016). Relationship between daily training load and psychometric status of professional soccer players. Res. Sports Med..

[39] Clemente F.M., Mendes B., Nikolaidis P.T., Calvete F., Carriço S., Owen A.L. (2017). Internal training load and its longitudinal relationship with seasonal player wellness in elite professional soccer. Physiol. Behav..

